# Of Mycelium and Men: Inherent Human Susceptibility to Fungal Diseases

**DOI:** 10.3390/pathogens12030456

**Published:** 2023-03-14

**Authors:** Donald C. Vinh

**Affiliations:** 1 Division of Infectious Diseases (Department of Medicine, McGill University Health Centre), Montreal, QC H4A 3J1, Canada; donald.vinh@mcgill.ca; 2 Division of Medical Microbiology, Division of Molecular Genetics-Immunology (Department of OptiLab, McGill University Health Centre), Montreal, QC H4A 3J1, Canada; 3 Centre of Excellence for Genetic Research in Infection and Immunity, Research Institute-McGill University Health Centre, Montreal, QC H4A 3J1, Canada

**Keywords:** inborn errors of immunity, primary immunodeficiency, fungi, mycoses, invasive fungal disease, superficial fungal disease

## Abstract

In medical mycology, the main context of disease is iatrogenic-based disease. However, historically, and occasionally, even today, fungal diseases affect humans with no obvious risk factors, sometimes in a spectacular fashion. The field of “inborn errors of immunity” (IEI) has deduced at least some of these previously enigmatic cases; accordingly, the discovery of single-gene disorders with penetrant clinical effects and their immunologic dissection have provided a framework with which to understand some of the key pathways mediating human susceptibility to mycoses. By extension, they have also enabled the identification of naturally occurring auto-antibodies to cytokines that phenocopy such susceptibility. This review provides a comprehensive update of IEI and autoantibodies that inherently predispose humans to various fungal diseases.

## 1. Introduction

Humans have lived intertwined with fungi since antiquity. Despite their virtues, fungi have also generated human diseases throughout history (antedating modern concepts of iatrogenically immunocompromised hosts), which were either mediated by toxins or manifesting as infection (mycoses). Invasive fungal disease (IFD) can either be superficial (i.e., invasive disease restricted to the mucosa, skin, and/or skin appendages) or deep-seated (invasive toward otherwise sterile tissue, e.g., visceral organs, fluid (e.g., blood), etc.). While the underlying basis for the host’s susceptibility to such fungal infections once remained obscure, a familial basis was observed in some cases, implying a hereditary component. The field of primary immunodeficiencies, now referred to as inborn errors of immunity (IEI), has enabled the discovery of some of these genetic elements. While fungal pathogenicity is the outcome of a complex interaction between a fungus, a host, and the environment, the field of medical mycology has focused on the former two in the context of iatrogenic immunosuppression, which is understandable because of its predominance in the clinical picture. On the other hand, defining the monogenic basis of human immunity to fungi in a natural state (i.e., in the absence of iatrogenic immunosuppression), which is performed through the field of IEI, can pinpoint critical molecular antifungal pathways. We review these disorders herein.

In the absence of anatomic defects, iatrogenic immunosuppression, or invasive procedures, fungal disease that is persistent, recurrent, and/or severe strongly suggests the presence of an underlying inborn error of immunity. However, the identification of this “fungal disease phenotype” is not a sufficient measure with which to label a person with an inborn error of immunity. In the purest sense, investigations identifying molecular lesions, which are mechanistically causative of an infection through immunological dysfunction, should be pursued. Dissecting the defective immunobiological lesions, even in singular patients, permits the proposition of immune pathways that may be critical for host antifungal defenses. The addition of each new member of this patient group then adds to or refines such pathways or perhaps even leads to the proposition of alternative ones, eventually leading to the development of pathophysiological constructs of human immunity to various fungi.

## 2. Framework Ascribing Genetic Immunodeficiency to Fungal Susceptibility

The ultimate purpose of defining IEI causally associated with IFDs is to develop a molecular immunological framework of human immunity to mycosis; such information may even shed light on fungal susceptibility in the more complex secondary immunodeficiencies. To achieve this, stringent criteria need to be explicitly considered to attribute penetrant, genetic susceptibility to fungal disease, and such criteria are proposed herein:

(1) The reported mycosis must have fulfilled standardized, diagnostic criteria of fungal disease, such as those defined by the European Organization for Research and Treatment of Cancer and Mycoses Study Group (EORTC-MSG) [1]. While initially formulated for IFD in iatrogenically immunocompromised patients, this framework provides provide a barometer for robust diagnosis. These criteria are intended to provide a strong deterrent against the misattribution of susceptibility to fungal disease based on colonization (rather than invasion), sample contamination, or non-specificity (e.g., based on certain serologic markers).
(2) The mycosis must have occurred prior to any medical intervention. Some IEI require exogenous immunosuppression for autoinflammatory or autoimmune features or transplantation for failing organ function or hematopoietic reasons. Some patients with IEI may require indwelling catheters (e.g., for intravenous access), which may become contaminated and then serve as a portal of systemic entry. In addition, some patients with IEI may require surgical intervention (which may or may not be related to their IEI), but which may become a nidus for IFD (e.g., intestinal leak/perforation). These states, in themselves, can be associated with an elevated risk of IFD. A fungal infection that develops only after the use of these treatments or in transplanted states cannot be definitively attributed to the underlying IEI exclusively, although such iatrogenic cases are conducive to the generation of hypotheses (especially if observed repeatedly and/or supported by mechanistic investigations).
(3) The mycosis must have occurred in an inborn error of immunity that was genetically identified and immunobiologically validated. This criterion is in place to avoid ascribing a fungal infection to a genetic variant of unproven significance.

The certainty of the association between a gene defect and fungal susceptibility is considered “strong” when a sufficient number of cases have been reported. Consistency in the clinical observation of susceptibility implies a persistent, inherent immunological basis. Given the rarity of IEI, and the potential ubiquitous or restricted geographic distribution of the implicated fungi with resulting variability in terms of individual exposure, a threshold of three unrelated cases is proposed and used herein. Alternatively, especially in cases where the associated inborn error of immunity or mycosis is extremely rare (e.g., with few cases or a single case reported), the demonstrable immunologic defect must be congruent with the immuno-pathophysiology for that specific mycosis previously shown in other IEI. Thus, a strong association is established when three or more unrelated cases have been reported or, in ultra-rare cases, there is pathophysiologic homogeneity for that mycosis in other IEI. Below this threshold, cases are considered “sporadic”, implying the need for additional reported cases or immuno-pathologic mechanisms to buttress the certainty of the association.

Collectively, these criteria aim to bolster the association of distinct mycoses with different IEI to more confidently identify underlying pathophysiological frameworks.

## 3. Candidiasis

Among the >200 defined *Candida* species, only a select few are specifically pathogenic to humans, with the foremost among these being *C. albicans* [2]. In a healthy state, *C. albicans* can be found as a commensal organism of the human microbiome, which, within the first year of life, colonizes the skin and gastrointestinal and/or genitourinary mucosae in as much as 70% of well individuals [3,4]. In this state, *C. albicans* likely reside in or above the dense layer of dead keratinocytes (stratum corneum) on the skin or in the mucus layer of mucosal surfaces. The acquisition of non-*albicans* species is less well characterized. Mucosal candidal colonization can be intermittent, with periods of loss of colonization, and can be influenced by environmental factors (e.g., tobacco), one’s physiological status (e.g., pre-menopausal), medical conditions (e.g., diabetes, HIV status, etc.), or medications (e.g., antibiotics, steroids, etc.) [5,6].

### 3.1. Chronic Mucocutaneous Candidiasis (CMC)

**Figure 1:**
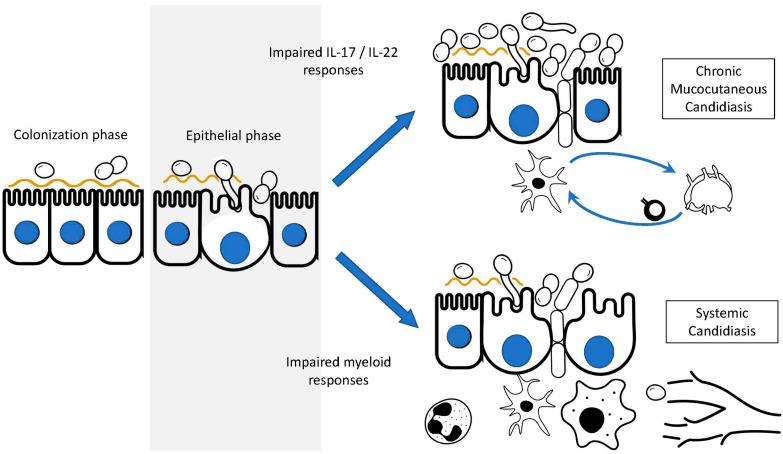
Human immunological framework mediating susceptibility to candidiasis. Colonization of the epithelial surface results in a commensal state. Transition to virulent forms and the elaboration of candidalysin promotes epithelial invasion. “Type 17” immunity, along with IL-22 responses, are key for superficial defenses, while myeloid responses guard against systemic disease. See text for details.

At these mucocutaneous barriers, *Candida* spp. can transition from colonizers to pathogens, leading to “superficial” disease, where invasion is restricted to the epithelial surfaces (Figure 1). Mucosal candidiasis manifests as one of several forms of oropharyngeal candidiasis (OPC, e.g., thrush/pseudomembranous OPC, erythematous lesions/atrophic OPC, and angular cheilitis), esophagitis, and/or vulvo-vaginitis. Cutaneous candidiasis can involve the skin (e.g., dermatitis, intertrigo, etc.) and/or nail structures (e.g., onychomycosis, paronychia, etc.). Mucocutaneous candidiasis can be acute or chronic. No specific time course formally distinguishes the “acute” from the “chronic” state; rather, clinical response to therapy, and thus, its recurrence or recalcitrance, is often used to guide the diagnosis of chronic mucocutaneous candidiasis (CMC).

The determinants of the transition from a commensal form to a pathogen at this interface include fungal virulence factors and alterations in the host defense mechanism. Selective defects in epithelial barrier integrity may permit CMC development based on its occurrence in the rare genodermatosis, keratitis, ichthyosis, and deafness (KID) syndrome [7,8,9,10,11,12,13,14,15,16,17]. KID syndrome, due to heterozygous mutations in the Gap junction beta-2 (*GJB2*) gene that encodes connexin-26 (Cx26), is complicated by chronic skin infections in approximately half of reported cases, with CMC being the most common [16,18,19]. Cx26 is a member of the connexins family, which oligomerize to form hemichannels in epidermal keratinocytes that either become functional channels at the plasma membrane or form gap junction channels by partnering with a hemichannel from an adjacent cell [20]. Cx26 controls dermal homeostasis by regulating keratinocyte differentiation and proliferation [21]. *GJB2* causal mutations precipitate the excessive opening of the Cx26 hemichannel, rendering it insensitive to the intra-epidermal variation in pH, and thus enabling the leakage of biomolecules that results in the increased proliferation of keratinocytes (hyperkeratosis) [20]. How such mutations confer susceptibility to CMC, whether at the level of the keratinocytes and/or through the impaired function of immunocytes, is unclear. Taki and colleagues have shown that human keratinocytes expressing a human mutation in Cx26 (p.D50N) demonstrated down-regulated expression of IL1A, IL-15, IL23R, CCL5, and TLR5 compared with wild-type keratinocytes, thus supporting the possibility that an immunologic defect intrinsic to epithelial cells may contribute to disease susceptibility [22]. On the other hand, older studies (prior to the discovery of mutant *GJB2* as the cause, and thus, in cases that were not genetically confirmed) have reported increased IgE levels [23,24], the absence of delayed-type hypersensitivity to *Candida* antigen despite CMC [25], impaired lymphocyte proliferative response to *Candida* in vitro [25], or defects in neutrophil chemotaxis [26]. Rerknimitr et al. reported a patient with CMC, KID syndrome (harboring the p.D50N mutation in *GJB2*), and the absence of S100^+^ cells (representing Langerhans cells (LC) in the epidermis) [15]. Since LC have been shown to drive Th17 cell responses [27], this finding could pathophysiologically connect KID syndrome, its CMC, and impaired IL-17 responses, although clearly more work is needed to confirm the paucity of LC in this disorder and mechanistically demonstrate “Type 17” immunodeficiency. Interestingly, no publications of other cases of genodermatosis (which were genetically confirmed) associated with an increased risk of CMC were identified. Thus, the hyperkeratosis of KID syndrome may contribute to candidal colonization, based on a similar phenomenon seen in psoriasis [28]; however, the absence of superficial candidiasis in the latter (along with psoriasis being an IL-17-potentiated process [29,30]) suggests that hyperkeratosis per se is insufficient to increase the risk of fungal disease. Intriguingly, KID syndrome can also predispose one to the dermatophyte *Trichophyton* sp. (see “Dermatophytosis” below).

The adhesion of *C. albicans* to epithelial cells triggers filamentous growth, i.e., the formation of pseudo-hyphae and hyphae, which is coupled with the elaboration of candidalysin, a fungal toxin that damages epithelial cell membranes [31]. The presence of candidalysin indicates a barrier breach, eliciting an epithelial host response consisting of antimicrobial peptides, chemokines, and cytokines, including IL-1α, IL-6, IL-8, G-CSF, and GM-CSF [31]. IEI saliently marked by CMC have been instrumental in defining the importance of the IL-17 response in coordinating susceptibility to superficial candidiasis (Table 1). CMC has long been recognized as a feature of severe combined immunodeficiency (SCID), a heterogeneous group of inherited disorders marked by T cell deficiency, with the concomitant deficiency of B- and/or NK-cells. Other, acquired defects of T cells (e.g., HIV/AIDS, transplant recipients, etc.) were also known to be significantly affected by CMC. Collectively, these conditions pinpoint the role of T cells in susceptibility to CMC. The importance of the CD4^+^ T helper cells subset characterized by the production of IL-17 (Th17 cells) was elucidated via dominant-negative STAT3 deficiency (DN-STAT3, previously called Autosomal-dominant hyper-IgE syndrome (AD-HIES) and originally termed “Job syndrome”), which is characterized by connective tissue anomalies (e.g., distinct facial features, hyperextensible joints, osteoporosis/bone fractures, scoliosis, retained primary teeth, and coronary and cerebral aneurysms), “cold” skin abscesses (boils) due to *S. aureus*, and CMC. Heterozygous mutations in the transcription factor STAT3 causing molecular dominant inhibition were discovered in 2007 [32]. Soon after, these mutations were found to impair the development of the adaptive Th17 cells, providing a key breakthrough in the understanding of the importance of IL-17-mediated responses for anti-candidal mucosal immunity [33].

STAT3 transduces the signaling effects of multiple cytokines, notably, IL-6, IL-10, and IL-23, thereby inducing the transcription of RORγt, which is critical for the development of Th17 cells, and the subsequent elaboration of IL-17 and IL-22. Loss-of-function (LOF) mutations in STAT3 thus impair Th17 differentiation, thereby crippling the IL-17 and IL-22 responses. Within the IL-17 family of cytokines, IL-17A and IL-17F have been best characterized in terms of fungal immunity; they function as homo- and hetero-dimers that signal through the IL-17 receptor (IL-17R), which is composed of IL-17RA and IL-17RC subunits [34,35]. IL-22 belongs to a distinct family of cytokines that acts on its cognate receptor (IL-20R), which is composed of the IL-22RA1 and IL-10RB subunits. IL-17R and IL-22R are expressed on keratinocytes. The distinct contributions of IL-17 and IL-22 to CMC immunity were shown in the elegant work conducted by the Gaffen lab [36]: IL-17A and IL-17F act on the supra-basal, post-mitotic epithelial cell layers (SEL), whereas IL-22 stimulates the basal, proliferative progenitor cells (BEL). IL-22’s activation of its cognate receptor on BEL and, consequently, its signaling through STAT3 lead to the replenishment of SEL that are responsive to IL-17. The IL-17 response, in turn, leads to a signature response of antimicrobial peptides, neutrophil-activating chemokines, and acute-phase response proteins [37].

Based on the compromised Th17 pathway in DN-STAT3 deficiency, the IL-17/IL-22 pathways were investigated in other IEI marked by CMC. Indeed, impaired IL-17 immunity emerged as a common theme in these disorders. Decreased IL-17 production was demonstrated in autosomal recessive (AR) IL-12p40 and IL12RB1 deficiency, which manifest principally with mycobacterial disease (see below), but in which 6–25% of patients are also affected with CMC [38,39,40]. In addition to diminished circulating IL-17-producing T cells, these patients also show decreased IL-22 production. Similarly, bi-allelic mutations in *ZNF341*, the transcription factor that regulates STAT3, lead to the loss of STAT3 and a phenocopy of AD-HIES/Job syndrome, including CMC with diminished IL-17 responses [41]. AR PGM3 deficiency is a congenital disorder of glycosylation that has occasionally been reported to manifest with CMC [42,43,44]. The precise immunological defect resulting from mutant PGM3 conferring susceptibility to CMC is not entirely understood, but slightly diminished Th17 levels have been described [42].

STAT1 gain-of-function (GOF) was originally discovered as a cause of CMC in 2011 for which poor production of IL-17 and IL-22 was observed [45,46]. It has since been identified as the most common genetic etiology of CMC. In addition to the latter infection, STAT1 GOF can also present with other mycoses (see below), non-fungal infections, and aneurysms, autoimmune diseases, and/or tumours [47]. Subsequently, AR RORγt deficiency, due to biallelic mutations in *RORC* (the transcription factor essential for Th17 lineage development), was identified [48]. AR CARD9 deficiency can be associated with CMC, although its more flagrant presentation is one of invasive fungal disease (see later). Loss of CARD9 function has variably been found in association with diminished, albeit not abolished, Th17 responses [49,50,51,52,53].

In addition to the preceding disorders that decrease IL-17 (and selectively IL-22) production, the distinctive feature of CMC and the principal of physiologic homogeneity led to the discovery of IEI that cause impaired responses to IL-17 due to either autosomal dominant (AD) mutations in the IL-17F cytokine [54], AR mutations in the IL-17 receptor subunits (IL17RA or IL17RC) [54,55,56,57,58], AR mutations in the adaptor molecule required for IL-17 receptor signaling (ACT1) [59,60,61,62,63], or AD mutations in the kinase essential for its signaling (JNK1) [64]. To date, no mutations compromising IL-22 signaling have been identified.

Beyond genetic lesions that compromise the production of IL-17/IL-22 or the response to IL-17, CMC can also result from the neutralization of these two cytokines, as shown in autoimmune polyendocrinopathy-candidiasis-ectodermal dystrophy (APECED, also called Autoimmune polyglandular syndrome type 1 [APS-1]), which is typically due to AR mutations in *AIRE*, although dominant-negative mutations have also been reported [65,66]. APECED patients classically present with CMC and endocrinopathies (hypoparathyroidism and adrenal insufficiency) but can also occasionally present autoimmune disease (e.g., enteritis, pneumonitis, etc.) or dental anomalies. The anti-cytokine auto-antibodies in APECED are present due to the loss of AIRE function, which leads to the loss of thymic regulation and, consequently, the escape of auto-reactive T cells.

Although CMC is clearly associated with a loss of IL-17 (+/− IL-22) immunity, the decreased levels of circulating Th17 cells identified represent adaptive (antigen-specific) T lymphocytes. Murine data have been used to identify the presence of mucus-residing γδ T cells, invariant natural killer T (iNKT) cells, innate lymphoid cell type 3 (ILC3) cells, and TCR+ ‘natural’ Th17 cells (nTh17), collectively referred to as “Type 17” cells, which are capable of producing IL-17 and IL-22 as well as GM-CSF [67,68]. Given their strategic location at the epithelial portal of entry, one or several of these cell types may be the immediate IL-17/IL-22 source in humans upon mucosal invasion by *Candida* sp. Further advances in characterizing tissue immunity on human mucocutaneous surfaces may help address this question and potentially guide the identification of novel genes responsible for these localized responses.

### 3.2. Invasive, “Deep-Seated” Candidiasis (IC)

Despite the persistent nature of CMC, with a clinically apparent fungal burden, CMC and IC are often mutually exclusive, implying the presence of distinct immunologic responses generated to control the respective disease and/or the compartmentalization of shared host defense pathways. In modern medicine, IC is most often the result of iatrogenic interventions or exogenous immunosuppression. In patients with acute leukemia, it has long been recognized that the risk of IC, as well as the severity of the fungal disease, correlate with the degree and duration of granulocytopenia, thus establishing circulating granulocyte levels as a proxy for risk of infection [69,70,71]. The risk also correlated with anti-leukemic agents and their toxicity to the gastrointestinal tract, with mucositis being an important determinant of invasive disease. This setting helped establish the importance of myeloid cells, particularly granulocytes (and primarily neutrophils at that), in the host defense against IC.

In distinction to the aforementioned paradigm, IC is rarely seen in IEI with myeloid defects. For example, “Congenital Neutropenia” (CN) refers to a heterogeneous group of disorders compromising neutrophil development. Despite the neutropenia (which can be profound), the risk of IC is not increased in these disorders [72]. Similarly, leukocyte adhesion deficiency (LAD) refers to a group of disorders in which mutant adhesion molecules (either integrins or selectins) impair leukocyte extravasation from blood vessels. Despite persistent neutrophilia, neutrophils are unable to mobilize to sites of infections, causing patients to develop recurrent/severe bacterial infections with a lack of pus formation. LAD patients have been considered at risk for fungal infections, but in reality, these manifest as intermittent episodes of mucosal candidiasis rather than IC [73,74,75,76,77]. Specific granule deficiency (SGD) is marked by neutrophils lacking secondary granule proteins along with an atypical nuclear morphology. SGD may be caused by both homozygous and heterozygous mutations in *CEBPE* [78]. Secondary (or specific) granules contain the antibacterial proteins lactoferrin, neutrophil gelatinase-associated lipocalin, cathelicidin, and lysozyme [79]. This functional defect results in recurrent bacterial infections but no reported increased risk for invasive fungal disease, including IC. Chronic granulomatous disease (CGD) is an inborn error of the phagocyte NADPH oxidase (phox) complex (see “Aspergillosis” section). The dysfunction of the phagocyte respiratory burst and the compromised generation of reactive oxygen species leads to recurrent bacterial and fungal infections as well as dysregulated granulomatous inflammation (e.g., colitis). Although patients with CGD are unambiguously at an increased risk for fungal infection, the mycoses tend to originate from mould (see the “Aspergillosis” section), and the risk of IC is remarkably rare. The rate of the latter may have been further influenced by the use of antifungal prophylaxis. Itraconazole prophylaxis was first shown to be effective in mitigating aspergillosis in 1994 [80,81]; subsequently, it was validated in a randomized clinical trial nearly a decade later [82]. Given that itraconazole prophylaxis is also effective against IC in other at-risk groups [83,84,85,86], it may have affected the modern risk of IC for patients with CGD. Nonetheless, IC was reported in CGD patients (diagnosed by nitroblue tetrazolium test) prior to the 1994 landmark findings [87,88,89,90] and subsequently [91,92]. Whether some of these cases may be iatrogenic (e.g., vascular-catheter-associated) is not clear, although in some reports, this does not seem to be the case. In distinction to IC, CGD is associated with an increased risk of systemic disease via infection with another yeast, *Trichosporon* [93,94,95,96,97]. Given the challenges in the antifungal management of trichosporonosis, biopsies for pan-microbiological culture should be performed. Collectively, these IEI indicate that myeloid defects do not automatically equate to an increased risk of IC.

In distinction to the absence of IC in the above IEI, there are rare reports of IC occurring in the absence of a known IEI. Specifically, there are historical descriptions of a fatal form of generalized moniliasis (the previous term for ‘candidiasis’) occurring in the absence of risk factors, including a fatal case involving the central nervous system [98]. Since then, several sporadic cases of spontaneous CNS candidiasis (sCNSc) have been reported, in which *Candida* sp. (primarily *C. albicans*) caused the infection of the brain and/or meninges without a preceding clinical intervention, underlying immunocompromising disorder, or apparent immunosuppression. Interestingly, many reports involved French-Canadian [99,100,101,102] and French [103,104,105] patients. With medical mycology focusing on the more-common IC/candidemia occurring in hematologic malignancies, transplants, and iatrogenesis, this historical syndrome had almost been forgotten until the discovery of AR CARD9 deficiency [49]. Although the initial work highlighted the susceptibility of CARD9-deficient patients to CMC, the clinical description of presumedly affected family members included one with candidal meningitis who developed seizures and hydrocephalus and then died, one with tumorous skull destruction, and one with candida meningo-encephalitis [49]. Drewniak and colleagues also reported a case of *C. dubliniesis* meningoencephalitis due to CARD9 deficiency [50]. The similarities of these CARD9-mutant cases to the historical sCNSc reports, along with the identification of a French-Canadian patient with sCNSc due to a c.T271C (p.Y91H) mutation [51], led to the recognition that biallelic mutations in CARD9 underlie sCNSc.

sCNSc is a form of chronic (subacute), disseminated candidiasis that is often the subject of clinical attention because of the involvement of the CNS (brain, meninges, and/or spinal cord) or related structures (e.g., the eye). However, non-neurological structures can also be affected, including the colon, liver, and bones [106,107,108,109]. The exact mechanism underlying susceptibility to invasive infection remains to be deciphered. However, one mechanism contributing to disease severity may be related to the impaired recruitment of neutrophils to sites of infection and/or impaired neutrophil clearance of yeast cells [50,110]. Mononuclear cell responses may also be defective (D. Vinh, manuscript in progress). These myeloid defects likely underlie the indolently progressive nature of reported cases, which can span years (presuming that these reports represent a single fungal clone that relapses). This presentation is in gross distinction from the acute forms of candidiasis seen in classical at-risk groups, which can be fulminant and is more reminiscent of the chronic, disseminated (hepatosplenic) candidiasis accompanying myeloid reconstitution following intensive cytoreductive chemotherapy [111,112]. Antifungal therapy may be effective in controlling sCNSc disease, although not invariably so. The cessation of antifungal therapy may be associated with a relapse of symptoms. It is unclear if the surgical resection of brain masses (often performed under the suspicion of a malignancy that is subsequently found to be an abscess) improves prognosis. Given that CARD9 deficiency has been associated with impaired peripheral blood mononuclear (PBMC) responses to *C. albicans* in vitro, including decreased fungus-induced GM-CSF production [51,52], and that large studies have shown that healthy controls distinctively produce GM-CSF in response to *C. albicans* (relative to other agonists) [113,114], adjuvant immunotherapy with recombinant human GM-CSF has been successfully used in some patients [51,52]. Similarly, given the associated myeloid defects, adjuvant G-CSF has also been effective [115,116]. Stem cell transplantation has also been attempted, wherein success led to clinical remission [117].

CARD9 deficiency has also been sporadically reported to predispose one to invasive disease caused by other yeast-like organisms, including *Saprochaete capitata* (previously known as *Geotrichum capitatum*) [118] and *Prototheca zopfii* (which is an achlorophyllous alga, not a fungi) [119]. Thus, it is important to carry out cultures and identification analyses to optimize antifungal therapy, and the reporting of such cases is informative.

## 4. Thermally Dimorphic Endemic Mycoses

Thermally dimorphic endemic fungi (TDEF) are saprophytes characterized by temperature-dependent morphological variation (i.e., they exist in a mould form at ambient temperature and assume a yeast-like phase at body temperature) and geographical restriction. These fungi include *Blastomyces*, *Coccidioides*, *Emergomyces*, *Histoplasma*, *Paracoccidioides*, *Sporothrix,* and *Talaromyces*. Updates on the microbiology of these fungi have been recently reviewed [120,121,122,123]. In their geographic niches, these fungi are environmentally distributed, and, based on previous seroprevalence or delayed-type hypersensitivity-based investigations, exposure is both frequent and generally asymptomatic or symptomatically self-limiting. On the other hand, some of these fungi are known to cause a variety of morbid syndromes in both overtly immunocompromised and apparently immunocompetent individuals. The field of IEI provides insight into the basis for the latter.

The first report of human blastomycosis in the Americas is attributed to Thomas Gilchrist, who presented the dermatological microscopic findings of a patient that was referred to him and, subsequently, detailed a second case of a chronically progressive, waxing-and-waning form of cutaneous blastomycosis [124]. Blastomycosis is most commonly caused by the *B. dermatitidis* complex (encompassing *B. dermatitidis* and *B. gilchristii*). Blastomycosis is said to be endemic to specific North American states and provinces that border the St. Lawrence River, the Great Lakes, the Mississippi River, and the Ohio River, although the geographic distribution of the causative fungi (and TDEF in general) is in flux owing to climate change and the human disruption of ecological niches [125,126,127,128]. Infection occurs via the inhalation of aerosolized conidia. The most common clinical syndromes of blastomycosis include acute pneumonia, chronic pneumonia, and extra-pulmonary (disseminated) disease, occurring in 25–40% of patients, which can involve the skin, musculoskeletal system, genitourinary tract, and/or the central nervous system [129].

Coccidioidomycosis (also known as San Joaquin Valley Fever, Valley fever, or Desert fever) is caused by two morphologically similar but genetically distinct fungi, *C. immitis* and *C. posadasii*. It was first described in 1888 by Alejandro Posadas and his mentor, Robert Wernicke, after their evaluation of Domingo Ezcurra, who had suffered from an indolently progressive but ultimately fatal form of the disease, and whose head is enshrined in a museum in Argentina [130]. *Coccidioides* is predominantly endemic in the Southwestern USA and Central and South America. Infection occurs following inhalation of arthroconidial spores. Coccidioidomycosis can manifest as various lung diseases (e.g., acute pneumonia, chronic progressive fibrocavitary pneumonia, pulmonary nodules, or cavities) or, in <1 to 5% of those infected, as a disseminated disease; the most common sites of involvement of the latter are the CNS, skin, musculoskeletal system, and lymph nodes.

Emergomycosis is caused by *Emergomyces* sp., whose genus was newly established in 2017 to include *Emmonsia*-like fungi [131]. Although disseminated disease has been reported in patients with advanced HIV or exogenous immunosuppression, no report of such mycosis associated with IEI has been identified.

In the Americas, histoplasmosis is typically caused by *H. capsulatum* (and its variants), whereas *H. duboisii* (restricted to Central and West Africa) causes African histoplasmosis. Although *H. capsulatum* was originally thought to be restricted to certain areas (e.g., central and eastern states surrounding the Ohio and Mississippi River Valleys, as well as the St. Lawrence River valley), it is now recognized that different species from the *Histoplasma* complex can be found worldwide [132]. Indeed, the first description of histoplasmosis by Samuel Darling in 1905 was of a disseminated, fatal form in a 27-year-old Martinique man presenting febrile vomiting and whose autopsy showed leptomeningitis with the involvement of the spleen and marrow [133]. Infection occurs via inhalation of small hyphal fragments and microconidia. Histoplasmosis can manifest as an acute pulmonary disease (varying from a mild/pauci-symptomatic form to severe/life-threatening forms); as complications associated with acute pulmonary disease that are thought to be immunologically mediated (e.g., erythema nodosum, arthritis, pericarditis, granulomatous, or fibrosing mediastinitis); as chronic cavitary pulmonary disease; or as disseminated disease [134].

Paracoccidioidomycosis (PCM), also known as South American blastomycosis, was first described by Adolpho Lutz in Brazil in 1908 who identified the disease in two patients with oral mucosal lesions and cervical lymphadenopathy [135]. Although PCM was attributed to *P. brasiliensis*, phylogenetic studies have identified various species within the *Paracoccidioides* species complex, although no distinction in clinical manifestations have been definitively established [136]. Infection is acquired by the inhalation of conidia. PCM is classified into distinct forms: The regressive form is a-/pauci-symptomatic, involving the lungs. The progressive disease can be acute/subacute (primarily with lymphadenopathy, hepatomegaly, and/or splenomegaly) or chronic (involving the lungs, and, usually, the skin, mucosa, and/or viscera, such as the adrenal glands or CNS) [136].

Sporotrichosis was first reported by Benjamin Schenck in 1898 who identified the disease through multiple refractory skin ulcers along the forearm lymphatics [137]. Unlike other TDEF, sporotrichosis more commonly occurs following the soft tissue traumatic implantation of infectious propagules from an environmental source; inhalation is a less frequent route of acquisition. Caused by members of the *S. schenckii* complex, sporotrichosis patients can present skin disease (cutaneous-lymphatic or nodular lymphangitis), disseminated disease (with visceral involvement), or extracutaneous presentations (e.g., CNS, musculoskeletal, or pulmonary) [138].

Talaromycosis (formerly Penicilliosis) is caused by *T. marneffei* (previously known as *P. marneffei*), the only TDEF within the *Talaromyces* genus that is pathogenic to humans. The fungus is notably endemic in South and Southeast Asia. It was first described by Gabriel Segretain and colleagues, who also reported the first human infected by the disease after accidental autoinoculation [139,140,141]. The first report of a naturally occurring infection was in the form of disseminated disease (wherein the fungus was isolated from a removed spleen) in a patient with Hodgkin’s disease [142]. Jayanetra and colleagues reported extensive disseminated disease in five patients, three of whom had no underlying disorder, causing death in three of the patients [143]. Infection follows inhalation of spores from the environment; although the bamboo rat is a known natural reservoir of the fungus, the exact environmental source, and how the bamboo rat contributes to transmission to humans, is unclear [144]. Given the fact that talaromycosis is a neglected tropical disease [145], our understanding of its clinical manifestations is limited [146]; thus, the prevalence of asymptomatic exposure is unclear. Pulmonary talaromycosis can include mass lesions, consolidation, cavities, effusion, or mediastinal lymphadenopathy. Patients with disseminated disease can present with skin lesions (which may be progressive and disfiguring), hepato/spleno-megaly, visceral infection, and/or CNS involvement.

**Figure 2:**
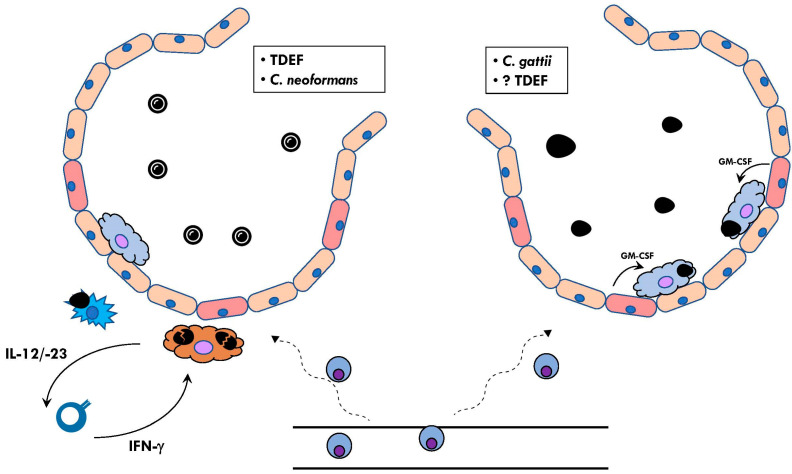
Immune pathways governing susceptibility to thermally dimorphic endemic fungi (TDEF) and cryptococci. Left: Inhalation of infectious propagules from TDEF or *C. neoformans* results in a cell-mediated immune response dependent on the production of IFN-γ and driven by IL-12/-23. Right: Host defense against *C. gattii* also relies on GM-CSF immunity. See text for details.

The general pathophysiology for these mycoses is similar to that for mycobacteria: the inhalation of the infectious propagules results in their deposition in the alveoli, where they are ingested by alveolar macrophages. Under the influence of body temperature, the propagules convert to their yeast-like form intracellularly, disseminating to regional lymph nodes. The combination of innate and adaptive immunity attempts to contain the infection, wherein the formation of granuloma occurs. T-cell-mediated immunity is critical for this containment. The failure of this process results in systematic, progressive dissemination. This similarity to the pathophysiology of mycobacteria has been central to identifying IEI associated with TDEF (Figure 2).

Genetic defects in the capacity to produce or respond to interferon-gamma (IFN-γ) have been the IEI most frequently reported associated with TDEF mycoses (Figure 2). Most of these fall in the collection of disorders referred to as Mendelian Susceptibility to Mycobacterial Disease (MSMD) [147,148,149]. While MSMDs were initially recognized for their distinct susceptibility to mycobacteria (particularly non-tuberculous (NTM) and occasionally *M. tuberculosis*) and *Salmonella* (predisposing victims to extra-intestinal salmonellosis) [38,39,150,151,152,153,154], they were also subsequently found in patients with otherwise unexplained disseminated TDEF infection, including histoplasmosis (IL12RB1 [153,155,156,157]; IFNGR1 [158]), coccidioidomycosis (IFNGR1 [159] and IL12RB1 [160]), and paracoccidioidomycosis (IL12RB1 [161]). These findings provided the framework of human immunity to TDEF, which was followed by the identification of other IEI congruent with defective IFN-γ-mediated immunity, including CD40L deficiency [162,163,164,165,166,167,168,169,170] (discussed further in the section on “Pneumocystis”), GATA2 deficiency [171], and STAT1 GOF [168,169,172,173].

GATA2 deficiency, due to heterozygous germline mutations in the GATA2 transcription factor, results in a wide spectrum of syndromes, including MonoMAC [174,175,176]; dendritic cell, monocyte, B, and NK lymphoid (DCML) deficiency [177]; familial myelodysplasia/acute myeloid leukemia (f MDS/AML) [178]; Emberger syndrome (entailing deafness, lymphedema, and MDS/AML) [179]; chronic neutropenia [180]; and Warts, Immunodeficiency, Lymphedema, and anogenital Dysplasia (WILD) syndrome [181,182]. The core feature of GATA2 deficiency involves defective myeloid homeostasis. Notably, the progressive development of monocytopenia and dendritic cell cytopenia, with variable degrees of lymphocytopenia, occurs. Intriguingly, despite the eventual (near)-complete absence of monocytes and DC, tissue macrophages and Langerhans cells (LC) are observed in this disease (e.g., in tissue biopsies). However, the development of opportunistic infections, such as disseminated disease with mycobacteria or with TDEF and the predisposition to pulmonary alveolar proteinosis (PAP, entailing the inability of alveolar macrophages to clear a surfactant from the airspace), suggests that such tissue macrophages/DC are functionally impaired. Whether that dysfunction is cell-intrinsic, as suggested in a mouse model [183], or due to a loss of cooperation from incoming monocytes/DC, or both, is not clear. One of the sentinel descriptions of GATA2 deficiency noted susceptibility to disseminated histoplasmosis, which was noted in several patients [174]. Subsequently, non-disseminated blastomycosis (pulmonary only, reportedly severe) was identified in a woman with GATA2 deficiency, although the trigger to identify this inborn error of immunity was not related to the fungal disease but rather the subsequent development of herpes-simplex-virus-induced Hemophagocytic lymphohistiocytosis (HLH) nine years later [171]. Variants in GATA2 have also been identified in a Brazilian cohort in association with histoplasmosis, paracoccidioidomycosis, and sporotrichosis [184]: Most of the variants were predicted in silico to be benign, while one was rare and classified as a variant of undetermined significance (minor allele frequency <0.01%; associated with paracoccidioidomycosis). Unfortunately, none of the variants were characterized molecularly or functionally. It is presumed that the progressive loss in the quantity and/or defective function of monocytes/DC compromise the effector arm of the IFN-γ axis, resulting in the failure of the containment of the fungal propagules and, subsequently, dissemination.

Mutations in STAT1 can result in a plethora of IEI. On the one hand, biallelic loss-of-function (LOF) mutations cause AR MSMD syndrome, with either partial or complete functional deficiency, resulting in susceptibility to mycobacteria and viruses [185,186,187]. Heterozygous LOF mutations can cause AD MSMD [188]. On the other hand, heterozygous GOF mutations cause “STAT1 GOF”, an inborn error of immunity that is distinct from MSMD and marked by susceptibility to TDEF [168,169,172,173] and other fungi (see “Chronic mucocutaneous candidiasis” and “Cryptococcus”). STAT1 GOF is a molecular gain-of-function mutation, that is, it allows for the normal phosphorylation of STAT1 but it is maintained for an abnormally long period of time (i.e., a prolonged phosphorylated state), leading to a loss of cellular function via a loss of responsiveness (tachyphylaxis) to repeated IFN-γ stimulation.

DN-STAT3 (see “Chronic Mucocutaneous Candidiasis” above) has been reported to be associated with TDEF. The resulting fungal disease in these patients, however, may be atypical in presentation (e.g., tongue, laryngeal, or gastrointestinal histoplasmosis) [189,190,191], although diffuse/visceral disease has also been reported (e.g., CNS coccidioidomycosis and disseminated talaromycosis) [192,193,194]. Whereas MSMD, CD40L, MSMD, and STAT1 GOF are unified by impaired IFN-γ immunity, the mechanism by which DN-STAT3 results in disseminated TDEF disease is not as well established. Diminished IFN-γ production has been identified, albeit with respect to *C. albicans* [195] or *A. fumigatus* [196]. Whether this defect is pertinent to TDEF remains to be demonstrated.

Intriguingly, mutations in T-cell-co-stimulatory molecules other than CD40L, notably those disrupting CD28/CD80 or CD86 (via recessive mutations in CD28 [197] or its essential signaling partners, RLTPR (also known as CARMIL2) [198,199,200,201,202,203,204,205] or CARD11 [206,207,208,209,210]) or ICOS/ICOSL interactions (via recessive mutations in either ICOS [211,212,213,214,215,216] or ICOSL [217,218]), have not been reported in association with TDEF. Whether this reflects stochasticity (i.e., a lack of exposure from geographic restriction among those with these rare diseases), or whether sufficient pathologic exposure has indeed occurred, but the respective molecules and T cell activation pathways are not critical for TDF host defenses, are questions that remain to be determined.

The recognition that defective production of/response to IFN-γ underlies at least some forms of severe TDEF mycoses led to the idea that supplementation with IFN-γ, at least for those with diminished production, may be a therapeutic strategy for the curing of infections. Indeed, IFN-γ immunotherapy, used adjunctively with antifungals, has been successful as a salvage therapy for refractory coccidioidomycosis in two IEI patients [159]. It has also been used successfully for this mycosis in patients without identified IEI [219,220,221], although one case presented modest improvement with IFN-γ, and the identification of a dysregulated Th2 response with the subsequent addition of dupilumab (to block IL-4- and IL-13-mediated Th2 responses) led to resolution [221]. Whether IFN-γ, either alone or with an inhibitor of dysregulated Th2 responses, may be successful for other TDEF remains to be reported.

Given the importance of the IFN-γ-immunity framework to TDEF, it was subsequently found that this pathway can be compromised by a distinct, non-monogenic process: the inherent presence of neutralizing auto-antibodies to IFN-γ (aa-IFN-γ). Again, the discovery of this immunologic defect underlying susceptibility to an infectious disease was first found in patients with a mycobacterial disease, namely, tuberculosis [222]. Subsequently, high-titer aa-IFN-γ concentrations were found in patients with no other immunocompromising condition in association with other severe disseminated infections, including NTM [223,224,225,226,227], and germane to TDEF, *H. capsulatum,* and T.(*P.*) *marneffei* [228,229,230,231,232,233,234,235,236,237,238,239,240,241,242] (as well as *Cryptococcus*, see below). aa-IFN-γ is an emerging, adult-onset form of immunodeficiency that is typically recognized in the fourth to sixth decades of life. An ethnic predisposition among Southeast Asians is noteworthy: these autoantibodies have been primarily reported among those of Filipino, Thai, Vietnamese, Japanese, and Chinese ancestry, with other ethnic groups less commonly affected [243,244]. A female predominance has been shown [241], as has a genetic association with the HLA alleles, DRB1*16:01 and DQB1*05:02 [245,246]. aa-IFN-γ inhibit STAT1 phosphorylation and IL-12 production. Thus, in patients (particularly women of Southeast Asian descent) with TDEF disease, particularly if severe or disseminated, aa-IFN-γ should be assessed. Of note, aa-IFN-γ were originally discovered in patients living with HIV (albeit without any clear association with infection susceptibility) [247], implying that HIV status and aa-IFN-γ are not mutually exclusive in terms of their existence and, possibly, compromising effects on immunity. The management of aa-IFN-γ has been challenging; standard broad-spectrum immunosuppressants (e.g., steroids) or immunomodulatory dosing of IVIG have been unsuccessful. Cyclophosphamide has been used with some success [240,248]. Given that this immunodeficiency is due to the presence of rogue autoantibodies, drugs specifically depleting B cells, such as Rituximab (anti-CD20) or Daratumumab (anti-CD38), have been reported to be effective in terms of reducing aa-IFN-γ levels and clearing infection [240,249].

## 5. Cryptococcosis

Cryptococcosis is principally caused by yeast members of the *C. neoformans* or *C. gattii* species complexes that have different geographic, microbiologically phenotypic, and genotypic characteristics [250,251]. Cryptococci also have a distinct host-range [252]: The *C. neoformans* species complex (herein referred to as “*C. neoformans*”) has traditionally been viewed as an opportunistic pathogen in various immunocompromising conditions, notably those of HIV and transplant recipients. Meanwhile, the *C. gattii* species complex (herein referred to as “*C. gattii*”) tends to cause disease in apparently immunocompetent people. The distinction between these species complexes is relatively recent and precludes interpretation of which cryptococci were identified in previous reports of IEI patients. Thus, the instances wherein *C. gattii* has been specifically identified from such cases will be indicated.

The molecularly defined IEI associated with severe or disseminated cryptococcosis strikingly overlap with those that predispose one to infection with TDEF, namely, MSMD [253,254,255], GATA2 [174,176], CD40L deficiency [163,256,257,258,259,260,261,262,263], STAT1 GOF [47,264,265], and DN-STAT3 [266,267]; these IEI have been detailed above. This overlap most likely reflects a comparable, if not homologous, immunopathophysiology (Figure 2). It also underlines a distinct feature of the genotype/phenotype correlations of IEI: the infection phenotype requires microbial exposure, which may be highly variable between individuals with the same lesioned gene, resulting in variable expressivity, at least of the mycoses. It should also be noted that cryptococcosis can be seen in “idiopathic CD4^+^ lymphocytopenia” (ICL), an immunologic phenotype that likely reflects the convergence of heterogeneous causes; in the absence of a clear genetic etiology, ICL is not included among these IEI, although clinicians should be aware of this possibility during their investigations.

As with the TDEF, aa-IFN-γ have also been found in patients with unexplained cryptococcosis [231,268,269]. The characteristics of aa-IFN-γ are detailed above (see “Thermally dimorphic endemic mycoses”). Compared to HIV-infected patients, these patients with aa-IFN-γ tended to be less likely to present with meningitis and were more likely to present with bone and/or joint and lung/pleura as well as skin infections [269].

In addition to aa-IFN-γ, high-titer, neutralizing auto-antibodies to GM-CSF (aa-GM-CSF) have been found in patients who were not overtly immunocompromised but who developed cryptococcal meningo-encephalitis that, distinctly, was associated with *C. gattii* [270,271]. aa-GM-CSF were first identified in patients with pulmonary alveolar proteinosis (PAP), a disorder characterized by dyspnea, alveolar infiltrates (determined radiologically), and a risk for progressive respiratory failure [272,273]. PAP results from the unregulated accumulation of a surfactant in the airways due to defective alveolar macrophage clearance [272]. PAP had been previously reported to be occasionally complicated by opportunistic infections, notably cryptococcosis (manifesting as the pulmonary form alone [274] or with systemic disease [275,276]), thus potentially linking aa-GM-CSF to cryptococcal susceptibility. GM-CSF is a pleiotropic cytokine involved in numerous functions, including the terminal differentiation of monocytes into mature alveolar macrophages, the differentiation of DC, and enhanced myeloid antimicrobial functions [277]. aa-GM-CSF inhibit the GM-CSF-induced STAT5 phosphorylation of peripheral blood mononuclear cells in vitro, although the exact pathophysiology of how they facilitate disseminated disease remains to be deciphered. Cryptococcosis (and other opportunistic infections) can precede, occur concomitantly with, or follow the clinical development of PAP. Intriguingly, the loss of the GM-CSF pathway is associated with susceptibility to another neurotropic infection (i.e., nocardiosis [278,279]), and the selective loss of GM-CSF response by PBMC to *C. albicans* may also contribute to candidal invasion of the CNS through CARD9 deficiency [51,52] (see “Invasive Candidiasis” above), suggesting a role for GM-CSF in systemic protection against CNS infection. The underlying basis for the development of aa-GM-CSF is unclear. In addition, while both aa-IFN-γ and aa-GM-CSF underlie susceptibility to “cryptococcosis”, it is unclear from the current published reports whether these auto-antibodies segregate with mutually exclusive susceptibility to the species complex (e.g., aa-IFN-γ to *C. neoformans* vs. aa-GM-CSF to *C. gattii* only) and whether these auto-antibodies can co-exist in patients.

Intriguingly, historical reports of patients with PAP complicated by histoplasmosis have also been reported [276,280], although no reports of aa-GM-CSF underlying human cases of histoplasmosis have been identified.

## 6. Pneumocystis

*Pneumocystis* was discovered in 1909 and, at the time, was thought to be a protozoan parasite (*Trypanasoma*); genomic analysis resulted in its subsequent re-classification as a fungus in 1988 [281]. Within this fungal genus are several species, each of which demonstrate strict target–host species specificity, that is, they are able to infect only one host species. Thus, humans can be infected with *Pneumocystis jirovecii* and are, consequently, the only source capable of infecting other humans, while the previously more popular term “*P. carinii*” is the formal name for the species that infects rats [282].

*P. jirovecii* pneumonia (Pjp) was initially reported in the late 1930s in Europe, with subsequent clusters reported in the 1940s and 1950s, as an unusual pulmonary infection characterized by a prominent infiltration of mononuclear cells thought to be plasma cells (hence the term “interstitial plasma cell pneumonia”) [283,284]. Strikingly, these epidemics affected premature and malnourished (“dystrophic”) infants, usually between 6 weeks and 4 months of age, for which mortality rates were as high as 50% and wherein autopsy series noted pneumothorax, mediastinal emphysema, and alveolar exudate filling the gray lungs. While the immunological cause of these Pjp outbreaks in infants remains unclear, Pjp was recognized as a potential cause of fatal infection in SCID through the Vetter family (with death of an older brother) and other reports [285,286,287], and clusters of Pjp in previously healthy adults in the 1980s heralded the HIV/AIDS epidemic [288,289,290]; this provided the first evidence that profound cell-mediated immunodeficiency was conducive to the development of disease.

Exposure to *P. jirovecii* seems to be common: based on serology data of healthy US children, two-thirds had detectable *Pneumocystis* antibodies by the age of 4 years [291]. Using molecular and serologic techniques, it was found that asymptomatic exposure was frequent in Chilean children [292]. Infection is thought to occur primarily following the person-to-person transmission of asci (cysts) via respiratory secretions. As with other fungi, *P. jirovecii* is not a professional pathogen, as asymptomatic colonization has been documented at high frequencies [293]. The adherence of trophozoites to alveolar type I pneumocytes initiates the infectious process, which progresses and may ultimately be fatal in immunocompromised patients. The exact immunopathophysiology of Pjp is a challenge to study owing to, among other things, the difficulty of culturing the organism. However, the natural history of Pjp in IEI underscores the importance of T cell immunity with respect to mitigating disease.

Combined immunodeficiency (CID) refers to a diverse group of IEI marked by defective cellular and humoral immunity. In its most severe form (Severe CID (SCID)), manifestations occur in infancy and typically include failure to thrive, recurrent infections, and opportunistic infections. Pjp can be a life-threatening infection in these IEI: prior to the routine use of prophylaxis, the overall Pjp infection rate in SCID was at least 27%, an estimated attack rate of at least 0.34 episodes per patient-year, with a true infection rate of 42% [286]. In some cases, Pjp may even be the sentinel manifestation of SCID [294]. SCID is fatal unless corrected early in life with hematopoietic stem cell transplantation (HSCT) or gene therapy (GT). In distinction to SCID, CID is survivable beyond infancy. While the associated defects may not be as profound as those in SCID, CID may also be associated with an increased risk of Pjp. Following the proposed criteria, the most frequently reported ones are described below.

HLA class II deficiency is an AR CID marked by the absence or greatly reduced expression of major histocompatibility class II molecules on the cell surface due to mutations in the MHC class II transactivator (CIITA), regulatory factor X-associated protein (RFXAP), regulatory factor X-5 (RFX5), and ankyrin-repeat-containing regulatory factor X (RFXANK) [295]. The major function of HLA class II molecules is to present antigens to T cells; hence, their expression is primarily restricted to thymic epithelial cells as well as professional antigen-presenting cells, namely, monocytes/macrophages, dendritic cells (DC), and B cells. Consequently, the loss of HLA class II function disturbs CD4^+^ T helper cell development and renders cell-mediated immunity defective. Similarly, Wiskott–Aldrich syndrome (WAS), which occurs due to X-linked recessive mutations in *WAS* that compromise the actin cytoskeleton of hematopoietic cells, is marked by CID, eczema, and microthrombocytopenia [296]. Mutant WAS protein (WASp) interferes with proper migration and antigen uptake, immunological synapse formation between APC and T cells, and T cell proliferation. Hyper-IgM syndrome (HIGM) is caused by various genetic lesions of the CD40/CD154 (also known as the CD40 ligand (CD40L)) axis. Since this axis enables cross-talk between activated CD4^+^ T lymphocytes (expressing CD40L) and B cells (constitutively expressing CD40), the loss of this function results in abnormal proliferation, class-switch recombination, and the somatic hypermutation of the latter lymphocytes, resulting in the impaired formation of antibody and memory cells [297]. Hence, patients classically have high IgM but low serum levels of IgG and IgA, leading to an increased risk for bacterial infections. Since the CD40/CD40L pathway also mediates co-stimulation between activated CD4^+^ T cells and CD40-expressing monocytes, macrophages, and dendritic cells, mutations in either CD40L (X-linked) or in CD40 (AR) also lead to opportunistic infections, notably Pjp [257,298,299,300,301,302,303,304,305,306,307,308,309,310]. Similarly, nuclear factor-kappa-B essential modulator (*NEMO/IKKγ*) [311,312,313], inhibitor of kappa light chain gene enhancer in B cells, alpha (*IκBα*) [314], or nuclear factor kappa-B subunit 1 (*NFKB1*) [315,316] are involved in CD40/CD40L signaling, and, when mutated, interfere with CD4^+^ T cell/APC co-stimulation, resulting in HIGM syndrome, for which there are reports of Pjp. On the other hand, several other genes involved in CD40 signaling but acting in a B-cell-intrinsic manner (e.g., activation-induced cytidine deaminase (*AICDA*), uracil–DNA glycosylase (*UNG*), phosphatidylinositol 3-kinase catalytic delta (*PIK3CD*), and phosphatidylinositol 3-kinase regulatory subunit 1 alpha (*PIK3R1*)) may cause HIGM but have not been reported in association with Pjp. IEI involving the Ikaros Zinc Finger (IkZF) family of transcription factors, specifically dominant negative mutations (DN) in *IKZF1* (encoding IKAROS) or in *IKZF3* (encoding AIOLOS), confer increased susceptibility to Pjp. IkZF transcriptionally regulates hematopoietic development. DN-IKZF1 deficiency impairs lymphocyte development, wherein CD4^+^ T cells that are primarily naïve and possess impaired function are developed [317]. Moreover, due to this deficiency, monocytes are dysfunctional [317]. DN-AIOLOS deficiency disrupts CD40/CD40L signaling [318]. These disorders suggest that the T–B cell interface (and its accompanying humoral immunity) is not critical for Pjp host defense. Rather, it suggests that an airway CD4^+^ T cell–APC interaction, or perhaps an airway CD4^+^ T cell–alveolar epithelial cell interaction, is central. As with the identification of Th17 defects and CMC, perhaps a novel Th subset (in the circulation or in the alveolar space) may be discovered that could shed light on Pjp immunopathology.

Pjp has been reported in individual cases of GATA2 [319], XLA [320,321], and DOCK8 [322] but without any mechanistic evidence attributing the mutant gene to disease. If these observations consistently recur, they may provide much-needed granular data on the mechanisms of susceptibility.

## 7. Aspergillosis

Aspergillosis refers to a group of infections caused by molds of the genus *Aspergillus* and can be divided into three broad categories [323,324]: allergic bronchopulmonary aspergillosis (ABPA); invasive aspergillosis (IA); and chronic pulmonary aspergillosis (CPA). CPA refers to a heterogeneous group of conditions that are either indolent (>3 months) in seemingly immunocompetent individuals who have underlying structural lung disease (including ‘fungus ball’/aspergilloma, an isolated aspergillosis nodule, or chronic cavitary pulmonary aspergillosis (CCPA)) or progressive (worsening < 3 months) in moderately immunocompromised patients (i.e., subacute invasive aspergillosis (SAIA), also called chronic necrotizing pulmonary aspergillosis (CNPA)). It has been estimated that humans inhale at least several hundred *Aspergillus* spores daily [325]. Thus, the inhalation of conidia into the lower respiratory tract explains why the lungs are the primary site of disease. However, extra-pulmonary disease can occur, either as a contiguous extension or dissemination from a pulmonary portal of entry or, presumably, by inoculation that is localized and/or of a high burden (e.g., in the sinuses, gastrointestinal tract, skin, and soft tissue). Among the genetic disorders associated with these aspergillosis syndromes, ABPA is primarily seen with CFTR mutations, while IA and CPA are largely seen in chronic granulomatous disease (CGD), autosomal dominant hyper-IgE (Job’s) syndrome, GATA2 deficiency, and CARD9 deficiency.

### 7.1. Allergic Broncho-Pulmonary Aspergillosis (ABPA)

ABPA is a hypersensitivity reaction precipitated by *A. fumigatus* in the tracheo-bronchial tree. ABPA is seen in patients with cystic fibrosis (CF), an autosomal recessive disorder caused by mutations in the cystic fibrosis transmembrane conductance regulatory (*CFTR*) gene [326]. Impaired CFTR function leads to chloride ion/water imbalance, resulting in the classical syndrome of excessive mucous viscosity at the respiratory epithelium, pancreatic insufficiency, and fertility issues, as well as an abnormally elevated sweat chloride test. Intriguingly, only 1–15% of CF patients develop ABPA [327], even though *Aspergillus* spp. is cultivable from sputum in as much as 57% of patients [327]. ABPA can also be seen in asthmatic patients. As in CF, only a subset of patients with asthma develop ABPA, and this selectivity seems to be associated with the mono-allelic carriage of a CFTR mutation, with an estimated odds ratio of ~10 [327]. Collectively, these findings suggest that ABPA is a CFTR-related disorder [328]. This intra-individual variability of ABPA has led to the identification of additional genes, in the context of underlying lung disease, that are associated with an increased risk of ABPA [329,330,331]. While the mechanism(s) by which defective CFTR function contributes to an exuberant allergic inflammatory response is not completely defined, it is possible that abnormal mucous composition from mono- or bi-allelic *CFTR* mutations trap *Aspergillus* conidia, allowing them to persist beyond the time period in which they would normally be cleared [332]. In addition, the CFTR protein is also expressed in lymphocytes, and CD4^+^ T cells bearing mutant CFTR demonstrate cytokine dysregulation with skewing towards a T helper type 2 (Th2) phenotype [333,334]. On the other hand, IEI with aberrantly excessive Th2 diathesis, such as CARD11, CARD14, CARML2, DOCK8, IKBKG, IL6R, and TBX21, have not (yet) been reported to lead to the development of ABPA, suggesting that Th2 skewing alone is not sufficient for the development of disease.

### 7.2. Invasive Aspergillosis and Chronic Pulmonary Aspergillosis

IA can be pulmonary, extra-pulmonary, or both. In reality, pulmonary disease spans the tempo spectrum of acute disease, SAIA, and CPA presented above, and these can all occur within the same patient, thereby discouraging the notion that they are mutually exclusive conditions or prototypical of one immunodeficiency compared to another.

Chronic granulomatous disease (CGD) is a primary phagocyte defect characterized by recurrent, life-threatening bacterial and fungal infections as well as dysregulated inflammation typically resulting in tissue granuloma formation. ‘Classical’ CGD is transmitted in either an X-linked (via *CYBB* encoding NOX2, previously known as gp91^phox^) or autosomal recessive (*NCF1*/p47^phox^; *NCF2*/p67^phox^; and *CYBA*/p22^phox^) manner [335]. Mutations in any of these subunits of the nicotinamide adenine dinucleotide phosphate (NAPDH) oxidase (phox) complex impair phagocyte respiratory burst and the generation of reactive oxygen species (ROS). Among IEI, CGD patients are distinctly susceptible to IA; at least 25–35% of these patients are affected [336,337,338]. IA in CGD can cause pulmonary disease. The clinical course can be chronic, spread in a contiguous fashion across anatomic planes, and is marked by the difficulty with it can be controlled with antifungals (the 3 “Cs”). Some of these features may be due to discrete species within the genus. For example, while *A. fumigatus* is considered the most common etiological agent of IA, it is not a singular species. Rather, moulds previously recognized as *A. fumigatus* now constitute a complex that is designated *Aspergillus* section *Fumigati* subgenus *Fumigati*. These sub-species (also referred to as “cryptic species”), in conjunction with host characteristics, may contribute to differences in clinical manifestations [336,339,340,341,342]. Additionally, CGD can lead to extra-pulmonary disease (e.g., paravertebral abscess, osteomyelitis, and CNS) [343,344,345]. Despite the severity of fungal disease, CGD patients may be a- or pauci-symptomatic, which is attributable to a lack of an inflammatory response, and may only be detected during screening exams (e.g., chest X-ray) or with a high index of clinical suspicion of non-specific symptoms.

CGD has some distinctive features of IA that are important. Firstly, CGD has unique susceptibility to infection with *A. nidulans*. Like *A. fumigatus*, *A. nidulans* is not recognized as representing a single species but rather a species complex; however, the impact of sub-species on the clinical trajectory of CGD is unclear [346]. Herein, they will be collectively referred to as the type species. *A. nidulans* is a poorly pathogenic mould that is better known for its use in studying genetics and cell biology or in the industrial synthesis of enzymes. However, in CGD, it is the second leading cause of IA, capable of causing both pulmonary and extra-pulmonary disease [347]. While the reason for its pathogenicity in CGD has long been enigmatic, the N-acetyl-galactosamine (GalNAc) content of this fungus’s exopolysaccharide, galactosaminogalactan (GAG), provides some insight [348]: the NADPH respiratory burst triggers the production of neutrophil extracellular traps (NETs), leading to hyphal damage and fungal killing. GAG confers mould resistance to NETs. *A. fumigatus* has abundant GAG expression, contributing to its resistance to neutrophil-mediated killing and thus its virulence. On the other hand, *A. nidulans* has greatly reduced GAG expression, owing to decreased GalNAc production, rendering it especially sensitive to the antifungal activity of NETs. The loss of the phagocyte oxidase complex in CGD leads to the loss of NET activity and the loss of *A. nidulans*’ non-pathogenicity. However, why *A. nidulans* is the second most common aspergilli causing disease in CGD [347], as opposed to other *Aspergillus* species, remains unclear; whether this simply reflects differences in environmental exposure (with *A. nidulans* being more ubiquitous than other species), or whether additional factors are at play, should be identified. A second distinguishing feature of IA in CGD is that serum galactomannan assays are insensitive in terms of diagnosing this fungal disease [349,350]; thus, a negative result does not exclude the possibility of IA (or moulds with cross-reactive galactomannans). Thirdly, in addition to CPA syndrome, CGD patients are also at risk for a fulminant syndrome called “mulch pneumonitis”, which is characterized by severe, acute, bilateral pneumonia with hypoxemia and respiratory failure and occurs following exposure to high levels of fungal spores from outdoor gardening activities [351]. Despite the administration of antifungals, anti-inflammatory steroids, and critical care support (e.g., mechanical ventilation, extracorporeal membrane oxygenation, etc.), mortality can be high, but not invariably so [351,352,353,354]. Of note, in some patients, ‘mulch pneumonitis’ was the sentinel presentation of CGD [351].

The different genotypes underlying CGD predispose one to different risk levels for IA, and the residual activity of the encoded NADPH–oxidase complex (rather than the genotype per se) determines this risk [355]. Interestingly, ROS production may also be associated with risk for IA in a non-CGD, hematopoietic stem cell transplant patient population; this risk relates to a genomic SNP that is involved in ROS production but apparently not involved in the phagocyte NADPH–oxidase complex [356]. On the other hand, IEI that are partial phenocopies of CGD have been identified, such as p40^phox^ deficiency (due to AR mutations in *NCF4*) [357] and protein kinase C δ (PKCδ deficiency due to AR mutations in *PRKCD*) [358,359,360]. Regarding p40^phox^ deficiency, patients have bacterial infections and hyperinflammation but no invasive fungal disease. The mutations underlying p40^phox^ deficiency are LOF or hypomorphic, and they variably impair ROS production in neutrophils and monocytes (ranging from normal to decreased, but at higher levels than in CGD) and normal ROS production in monocyte-derived macrophages (moDM). PKCδ deficiency is primarily clinically marked by recurrent bacterial infections (involving the lungs, lymph nodes, and GI tract); no IA has yet been reported. In PKCδ deficiency, ROS production by neutrophils and monocytes mirrors that of p40^phox^ deficiency, although in distinction to the latter, PKCδ deficient-moDM have very low levels of ROS production. On the other hand, MSMD due to hypomorphic mutation in CYBB (MSMD^CYBB^) manifests as an X-linked susceptibility to mycobacteria exclusively rather than the range of bacterial and fungal infections of CGD [361]. The hypomorphic mutations of MSMD^CYBB^ molecularly distinguish it from the LOF CYBB mutations causing CGD. Moreover, at the cellular level, MSMD^CYBB^ is characterized by normal respiratory burst in neutrophils or monocytes ex vivo, but impaired respiratory burst selectively in moDM and patient-derived EBV-immortalized lymphoblastoid cell lines (LCL). Collectively, these IEI indicate that cell-specific ROS generation in a fungal morphotype-specific manner may contribute to susceptibility to IA. When focused on the respiratory–epithelial interface, inhaled conidia are thought to initially encounter alveolar macrophages, which use (at least) the phagocyte NADPH oxidase complex to clear the spores. In reality, the cellular components of the alveoli include type I and II pneumocytes as well as alveolar macrophages, and the alveolar surface is primary covered by type I pneumocytes (~90%) and type II pneumocytes (5–10%) [362]. Thus, the host cells that the inhaled conidia will most likely encounter first will be pneumocytes; indeed, in rats, such cells express the components of the phagocyte NADPH oxidase complex, with substantially higher levels of NOX2 (gp91^phox^), p22^phox^, p67^phox^, p47^phox^, and p40^phox^ subunits as well as Rac1 in type I pneumocytes than in type II [363]. Although human bronchial epithelial cells generate ROS in response to *A. fumigatus* conidia [364], whether the phagocyte NADPH–oxidase complex is operational in pneumocytes’ defenses against aspergilli has not, to the best of our knowledge, been studied. However, if it were operational, it would be defective in ‘classic CGD’, owing to the germline nature of the mutations, but variably so—if not at all—in p40^phox^ deficiency, PKCδ deficiency, and MSMD^CYBB^. It is potentially this junction that is critical in aspergilli pathogenesis. Failure to clear conidia (ostensibly by pneumocytes) results in their swelling and germination in the alveoli. Alveolar macrophages patrolling the airspace attempt to phagocytose and destroy the conidia. Local inflammatory responses recruit myeloid cells, particularly neutrophils, that are able to phagocytose resting and swollen conidia and kill mould hyphae (e.g., with NETs). When the phagocyte NADPH–oxidase complex is simultaneously defective in each of these components (pneumocytes, macrophages, and neutrophils), IA ensues. However, in IEI where there is a limited, cell-restricted defect in respiratory burst, IA does not transpire. Lastly, primary ciliary dyskinesia (PCD) is a heterogeneous group of inborn errors of respiratory cilia marked by recurrent airway infections and, occasionally, with airway colonization by *Aspergillus* and the development of ABPA [365,366,367,368]. Strikingly, PCD patients have not shown increased rates of IA, suggesting that defective mucociliary clearance does not sufficiently impair anti-aspergilli immunity.

The high rate of IA in CGD patients led to the initial study on antifungal prophylaxis with itraconazole, demonstrating a reduction in rate of IA to one-third [80]. This effect was later confirmed in a randomized clinical trial [82]. Despite this, a breakthrough fungal disease had been observed, which, in some cases, was attributable to pharmacologic issues (e.g., adherence, absorption, etc.) [369]. Additionally, CGD patients are at risk for infection with other moulds, albeit less frequently, including other hyalohyphomycetes (e.g., *Paecilomyces* [370,371,372] and the morphologically similar *Rasamsonia* [373] and *Fusarium* [374]) and basidiomycete mushrooms (e.g., *Phellinus* [375,376,377]). Thus, some patients may receive voriconazole or posaconazole prophylaxis. The diversity of moulds capable of causing disease in CGD should prompt the need for tissue sampling for microbiological investigations. Owing to the refractory nature of IA or some of the other mycoses, granulocyte infusions have been attempted successfully [378,379,380], although there may be some reporting bias. Granulocyte infusions can also be given as a bridge to HSCT [381,382], although there is a risk of allo-immunization with subsequent graft failure [383], or given post-HSCT as a bridge to myeloid reconstitution [384]. HSCT with concomitant antifungal therapy, without granulocyte infusions, has also been successful [385].

Other IEI with a consistently reported risk for IA include DN-STAT3 (AD-HIES, or Job’s syndrome), IL6ST deficiency, CARD9 deficiency, and GATA2 deficiency. In DN-STAT3 (see the “Candidiasis” section presented above), IA is typically restricted to pulmonary disease in people with abnormal lung parenchyma (e.g., pneumatoceles, cysts, and bronchiectasis) [192,386,387,388,389,390,391,392]. This observation, along with the finding that myeloid cells from such patients equally inhibit *A. fumigatus* in vitro, suggests that, in this syndrome, STAT3 mutations confer susceptibility to IA through defects at the level of the lung epithelium [192]. Whether this reflects a defect in alveolar epithelial homeostasis (e.g., STAT3 regulates pneumocyte regeneration [393]) and/or a pneumocyte-intrinsic host defense defect remains to be fully elucidated. The IA of DN-STAT3 tends to behave in a manner similar to that of CPA, although lung erosion with pleural involvement, hemoptysis, or the dysfunction of lung function are serious concerns. Unfortunately, despite prolonged antifungal therapy and/or surgical resection, the clinical outcomes are sub-optimal [192,391]. Heterozygous, dominant negative mutations in IL6ST (DN-IL6ST) can also cause AD-HIES syndrome [394]. As with DN-STAT3, DN-IL6ST is associated with IA, manifesting primarily as a CPA, in the context of abnormal lung parenchyma. *IL6ST* encodes GP130, the shared transmembrane signalling subunit of the IL-6-receptor (IL6R) family. Intriguingly, two other IEI, AR IL6ST deficiency [395,396,397] and AR IL6R deficiency [398], can lead to clinical syndromes with phenotypic overlaps with that of DN-STAT3 or DN-16ST. However, IA has not been reported in the two former IEI; this may be due to the censorship of data, as these patients were diagnosed at a younger age than DN-STAT3 and with fewer pulmonary parenchyma abnormalities (in some patients, no anomalies were reported). In CARD9 deficiency (see the “Candidiasis” section presented above), both CPA [399] and extra-pulmonary disease without concomitant lung involvement (e.g., intra-abdominal, CNS, and cutaneous) [108,400] have been observed. As with sCNSc (see the “Candidiasis” section presented above), the extra-pulmonary aspergillosis of CARD9 deficiency is associated with a paucity of neutrophils on tissue histopathology, despite visible fungus, contributing to development of disease, although the underlying susceptibility at these unusual anatomical sites remains obscure. GATA2 deficiency has been reported in association with IA both prior to chemotherapy or HSCT for MDS/AML and, more commonly, during or following the execution of these treatment modalities [174,180,401,402]; as explained in the proposed criteria, the latter state renders the distinction between infection due to iatrogenesis vs. inherent susceptibility inconclusive. It may also be that GATA2 mutations in the context of these treatment modalities confer a heightened risk for IA relative to those who do not harbor mutations [403]. In the pre-chemotherapy/HSCT state, pulmonary aspergillosis can be seen, and the risk is particularly increased if there is pulmonary alveolar proteinosis (PAP) [404]. Whether the IA reflects the impaired clearance of conidia due to accumulated proteinaceous surfactants or signifies impaired alveolar host defenses remains unclear.

While other IEI, particularly those implicating neutrophil dysfunction, may be considered to dispose one to the risk of IA, few reports fulfilling the above criteria have been identified. For example, previous reviews reported that congenital neutropenia or leukocyte adhesion deficiency predispose one to IA, and this has been reiterated in recent reviews. However, when probing the cited references to focus on published clinical cases, there is a dearth of such reports, which spans various IEI/neutrophil dysfunctions [405,406,407], or the data are published as registries [408] without the granularity of details to address the issues raised in the proposed criteria. Similarly, IA has been reported in STAT1 GOF, but with no details provided [47]. Thus, additional reports with relevant information are strongly needed to revisit these associations. Nonetheless, if these IEI are associated with IA (or, in the case of IEI marked by neutrophil dysfunction, with other IFD), such an association appears to occur relatively infrequently.

## 8. Dermatophytosis

Dermatophytes are hyalinated moulds of the Arthrodermataceae family with the capacity to invade keratinized tissue (i.e., skin, hair, and nails), causing “dermatophytosis” (colloquially referred to as “ringworm” or “tinea”). Dermatophytes were traditionally classified into three genera: *Trichophyton*, *Epidermophyton*, and *Microsporum*. Phylogenomic studies have since revised the taxonomy into seven genera: *Trichophyton*, *Epidermophyton*, and *Microsporum*, as well as *Paraphyton*, *Lophophyton*, *Arthroderma*, and *Nannizzia* [409,410]. Infection is acquired by the deposition of spores from humans, animals, or soil, accounting for anthrophilic, zoophilic, or geophilic spread, respectively. In immunocompetent individuals, infection is restricted to the stratum corneum, the outermost layer of the epidermis that contains a thickened layer of dead keratinocytes.

**Figure 3:**
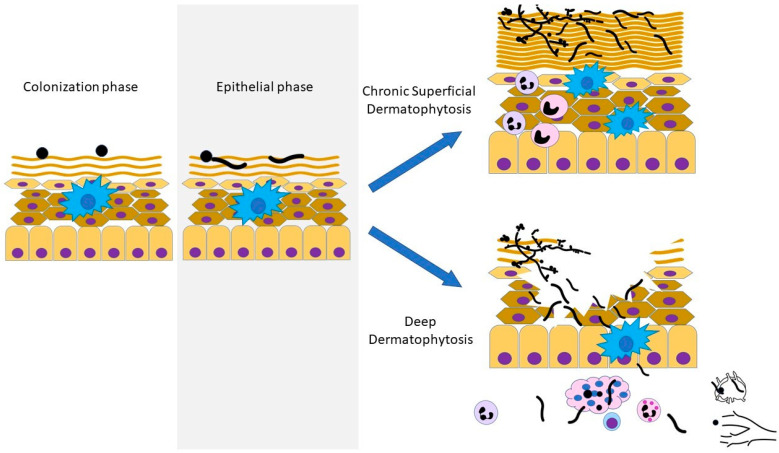
Host defense against dermatophytes. Dermatophytes can colonize keratinized surfaces. Disorders of keratin homeostasis may be associated with increased risk of developing chronic superficial dermatophytosis, wherein disease is limited to the skin. In rare instances, CARD9 deficiency allows dermatophytes to permeate deeper structures, wherein systemic dissemination can also occur. See text for details.

Despite their broad distribution and their affinity for a ubiquitous compound (keratin), dermatophytes are not professional pathogens. Previous serial sampling data have shown that these moulds can be isolated from humans for prolonged periods of time (months to years) in the absence of symptomatic infection [411]. Similarly, intra-familial transmission has been noted to be non-uniform. Thus, dermatophytes, like other microbes, demonstrate a spectrum of symptoms, ranging from asymptomatic infection and active disease to chronic disease. Chronic dermatophytosis has been previously defined as disease that is refractory to therapy, relapsing once treatment is stopped, and/or progressive [412]. Based on the genetic disorders described below, one could further separate ‘chronic dermatophytosis’ into two distinct chronic syndromes: one that manifests as a relapsing or recalcitrant, superficial disease (herein termed “chronic superficial dermatophytosis” [CSD]), and the other marked by superficial disease associated with invasion into deeper, non-keratinized tissue (termed “deep dermatophytosis” (DD)) (Figure 3).

As with CMC, genetic disorders of epithelial integrity predispose one to CSD. Although numerous cases of various ichthyosis disorders (e.g., ichthyosis vulgaris, lamellar ichthyosis, etc.) associated with dermatophytosis have been reported, dermatological diagnoses have been made clinically or, occasionally, via skin biopsy and without genetic confirmation [413,414,415,416,417,418,419,420,421,422,423,424,425,426,427]; following the proposed criteria above, these cases are not considered as definitive in this review. On the other hand, KID syndrome (due to dominant mutations in *GJB2*—which encodes Cx26—and conferring susceptibility to CMC (see above)) has also been associated with an increased risk for extensive CSD [10,12,19,428]. Some forms of palmoplantar keratoderma (PPK; also called ‘tylosis’) were also noted to be associated with increased rates of CSD [412,429]. PPK is a heterogeneous group of disorders characterized by the thickening (hyperkeratosis) of the palmar and plantar skin. Autosomal-dominant diffuse PPK can occur due to dominant negative mutations in keratin 9 (*KRT9*) [430], keratin 1 (*KRT1*) [431], or aquaporin 5 (*AQP5*) [432]. In previous studies from Norbotten, Sweden, the reported prevalence of associated dermatophytosis varied between 35 to 65% [433]. Keratins are a multi-gene family of proteins that form intermediate filaments, which are the major cytoskeletal proteins in epithelial cells. Mutations in *KRT9* function through dominant negative interference with the assembly of these filaments, and their specific contributions to PPK are due to the exclusive expression of KRT9 in the epidermal keratinocytes of the palms and soles [434]. It has been proposed that mutations in *KRT1* interfere with keratin intermediate filament interactions with other structures (e.g., desmosomes) or with the cross-linking of the cornified envelope [431]. AQP5 is expressed in the granular layer of the skin; PPK-causing GOF mutations disrupt keratinocyte water barrier function in the epidermis of the palms and soles [432,435]. Thus, selective defects in the barrier functions of the skin may confer susceptibility to CSD.

CSD, which primarily occurs due to *Trichophyton* spp. or *Microsporon* spp., has also been reported in STAT1 GOF [45,47]. This inborn error of immunity also predisposes one to CMC (see above). Whether this reflects the convergence of host defense pathways to mitigate these two superficial mycoses, or whether STAT1 GOF has a distinct mechanism of susceptibility that implicates skin barrier function, remains to be explored.

Sentinel descriptions of chronic, progressively invasive dermatophytosis were made in the late 19th century when Majocchi described granulomatous dermal disease caused by *Trichophyton* [436]. Subsequently, rare cases were reported of chronic generalized trichophytosis involving the lymph nodes, muscles, bones, brain, or other viscera, including familial cases. One of the earliest documented cases of a family with fatal dermatophytosis was reported in 1975 involving a young Algerian girl who had extensive cutaneous disease at 14 years of age that progressed over 8 years [437]. She had two brothers, each of whom died at 11 and 13 years old, respectively, from a dermatophytic disease involving the skin, hair, and viscera that was caused by *T. schönleini*, which was also identified from her skin lesions. Two other brothers were being treated for dermatophytosis (no further details were provided in this case), leading to the suspicion of a genetic immunodeficiency with susceptibility to dermatophytes. Further immunologic investigations at the time revealed intact humoral immunity but variably depressed cell-mediated immunity [438]. Indeed, Marill observed three key characteristics of what he termed “maladie dermatophytique” (French for “dermatophytic disease”) [439]: familial predisposition; deficiency of cell-mediated immunity; and unusual severity, with one patient developing a cerebral abscess and the other patients having died from the illness.

The breakthrough in understanding the underlying basis for this disorder came with the discovery of biallelic mutations in *CARD9* that were found in 17 patients of Algerian, Tunisian, or Moroccan descent with deep dermatophytosis (DD) [440]. Thus, AR CARD9 deficiency not only predisposes one to CMC or extra-pulmonary aspergillosis (discussed above) but to DD as well. The vast majority of cases are caused by *Trichophyton* sp. (e.g., *T. rubrum*, *T. violaceum*, and *T. mentagrophytes*), although *Microsporum* sp. [441] may occasionally be the culprit. DD can be quite destructive, with painful, non-healing cutaneous ulcers that often fail to resolve with antifungal therapy or risk relapsing once antifungal therapy is stopped. Importantly, DD can also manifest with lymphadenitis or visceral abscesses, from which fungal elements can be seen on biopsy and the dermatophyte is cultivable from tissue.

The mechanism(s) by which CARD9 mutations lead to seemingly disparate fungal diseases is not clear. Although CARD9 LOF mutations do impair IL-17 responses, the absence of DD in other IEI marked by IL-17 deficiency (which manifest with CMC, as presented above), other than a singular report of DD in a patient harboring a DN-STAT3 mutation [442], implies that more specific immunologic mechanisms need to be deciphered to understand how CARD9 mediates dermatophytic disease.

## 9. Pheohyphomycosis (Dematiaceous Fungi)

Pheohyphomycetes (dematiaceous fungi) are environmentally distributed moulds that are darkly pigmented due to the presence of melanin in the fungal cell wall. The host–fungal interaction can result in three human syndromes: eumycotic mycetoma, chromoblastomycosis, and phaeohyphomycosis [443]. Eumycotic mycetoma is a chronic cutaneous/subcutaneous infection characterized by the triad of ‘tumefaction’ (i.e., the indolent appearance of inflammatory nodules/mass with fibrosis); the penetration of sinuses or fistulae into deep tissue; and presence of granules in tissue or discharge [444]. Chromoblastomycosis is a chronic cutaneous/subcutaneous infection characterized by the presence of fungal muriform cells (also called ‘sclerotic bodies’ or ‘copper pennies) in tissue. Pheohyphomycosis refers to infections caused by these dematiaceous fungi that are histologically distinct from the two other syndromes due to the presence of yeast-like cells and filamentous growth in tissue; additionally, disease is not necessarily restricted to cutaneous/subcutaneous organs but can be disseminated (including with the involvement of CNS). Importantly, the manifestation of the clinical syndrome resulting from the infection with these fungi is driven by the immune status of the patient.

The identification of dematiaceous fungi in a diagnostic microbiology laboratory traditionally involves the assessment of the sample fungus’s growth rate, its colony and microscopic morphological characteristics, and the execution of biochemical tests. Since different medium and culture conditions can lead to different morphologic forms for a given fungus, this approach has led to some confusion about nomenclature. Genomically based identification has emerged as a more accurate method of identification.

Superficial disease due to dematiaceous fungi is commonly restricted to global regions of warmer climates and lower latitudes. Unfortunately, this has hampered our understanding of underlying fungal pathophysiology. The classical paradigm has been that these fungi are widespread in the environment, including in soil and plants, and that trauma, even minor forms, account for the ensuing disease. Of course, this explanation is both simplistic and overlooks a clear knowledge gap: minor trauma must occur quite frequently in the respective socio-economic context, yet disease does not. A single case of CARD9 deficiency causing chromoblastomycosis due to *Phialophora expanda* (within the *P. verrucosa* complex) has been reported [445]. Although an individual case, the authors elegantly buttressed this report with a mouse model replicating the disease and strongly suggested that CARD9 should be assessed in more cases of chromoblastomycosis.

Pheohyphomycosis has also been known for a long time but has remained enigmatic. Indeed, Marill was among the first to describe a “deep dematiaceous disease” due to *Hendersonula toruloidea* (a fungus at the time known to cause disease in fruit trees, which is now called *Neoscytalidium dimidiatum*) in an Algerian 30-year-old male who had, intriguingly, been diagnosed with a “deep dermatophytosis” at the age of 15 year and treated intermittently with griseofulvin [446]. Based on his investigations at the time, Marill suspected an immunological disorder. Nearly 35 years later, the first evidence to support his hypothesis was confirmed with the identification of AR CARD9 deficiency in four patients with pheohyphomycosis due to *P. verrucosa* [447]. This inborn error of immunity has since been identified in pheohyphomycosis due to *Exophiala* [448,449,450,451], other *Phialophora* species [452], *Aureobasidium* [453], *Alternaria* [454,455], and *Exserohilum* [456], as well as more esoteric dematiaceous fungi, including *Pallidocercospora crystallina*, *Ochroconis musae,* and *Corynespora cassiicola* [449,457,458,459]. Intriguingly, disseminated infection with *C. cassiicola* (with extension into the CNS) has also been reported in a patient with compound heterozygous mutations in *CLEC7A* (encoding Dectin-1, which is a fungal pattern recognition receptor that signals through CARD9) [460]. Other pheohyphomyces have also been reported with single heterozygous variants in *CLEC7A* or *CARD9*, although with distinct fungal etiologies than those for AR CARD9 deficiency [460]. Whether this distinction ostensibly reflects other genetic loci capable of influencing susceptibility to infection or severity to dematiaceous fungi or reflects inherent differences in fungal pathogenicity, or both, remains to be determined.

The management of pheohyphomycosis associated with AR CARD9 deficiency is quite heterogeneous, and clinical outcomes are not consistently reported. Various antifungals, either administered alone or in combination, have been used either as a sole therapy or in conjunction with surgical resection. This fungal disease is not invariably fatal, although relapses may occur. No reports of adjunctive cytokine therapy, as in AR CARD9 deficiency with invasive candidiasis (see above section), have been identified.

## 10. Mucormycosis

Fungi of the Mucorales order have aseptate (or pauci-septated), ribbon-like hyphae. The classical, acquired predisposing factors for infection with this fungus include neutropenia associated with chemotherapy for hematological malignancy, diabetes mellitus (particularly with poor glucose control and ketoacidosis), trauma, and iron chelation therapy. On the other hand, Mucormycosis has not been stereotypic or pathognomonic with respect to any single IEI. In CGD, spontaneous mucormycosis is exceedingly rare [343,461]. Most reported cases only occurred in patients receiving significant immunosuppression for several weeks [462], following HSCT [463,464], complicating anti-TNF-α treatment [465], or following invasive procedures [466,467]. However, an isolated case has been reported in the absence of immunosuppression [468]. The aggregate of these cases would suggest that defects in the phagocyte NADPH–oxidase system are not sufficient in terms of susceptibility to these moulds. There have also been reports of mucormycosis linked to AR CARD9 deficiency, wherein every case corresponded to exclusively cutaneous disease and was caused by *Mucor irregularis* (formerly *Rhizomucor variabilis* var. *variabilis*) [469,470,471]. Interestingly, *M. irregularis* is distinct among the Mucorales due to its tendency to cause chronic cutaneous/subcutaneous infection without angio-invasion and deeper dissemination, although the disease can be mutilating [472]. What is equally interesting is that all three reported CARD9 deficiency cases occurred in Chinese patients, and cases of the chronic skin disease caused by *M. irregularis* have exclusively been reported in Asia, particularly China [472], perhaps reflecting some aspect of selective evolutionary pressures. Mucormycosis has also been singularly reported in association with GATA2 deficiency (although it is not clear if this case occurred during chemotherapy or HSCT) [473] and STAT1 GOF [474]. Clearly, more reports of native mucormycosis in IEI would significantly guide our understanding of human immunity to these moulds.

## 11. Conclusions

Fungal diseases reflect an underlying immunological deficiency. In the absence of an obvious medical condition or exogenous immunosuppression, patients with persistent superficial mycoses or those with invasive fungal disease should be evaluated for an underlying inborn error of immunity. Genomically based investigations combined with the functional validation of identified rare genetic variants and the characterization of their impacts on immunological functions and host–fungal interactions will further our understanding of human immunity against fungi. These insights will identify molecular targets that will pave the way for novel anti-fungal immunotherapies.

## Figures and Tables

**Table 1:** IEI with increased susceptibility to CMC.

Inborn Error of Immunity	Gene	Mode of Inheritance	Comment
Decreased IL-17 production			
DN-STAT3 (Job’s syndrome)	STAT3	AD	Decreased Th17 responses
IL12p40 deficiency	IL12B	AR	MSMD
IL12Rβ1 deficiency	IL12RB1	AR	MSMD
ZNF341 deficiency	ZNFR341	AR	Phenocopy of DN-STAT3 (Job’s syndrome)
PGM3 deficiency	PGM3	AR	Congenital disorder of glycosylation
STAT1 GOF	STAT1	AD	Associated with various infections and aneurysms, autoimmune disease, and/or tumours
CARD9 deficiency	CARD9	AR	Variably decreased Th17 responses
Decreased IL-17 response			
IL-17F deficiency	IL17F	AD	Afflicted patients may also be at risk for *S. aureus* skin and soft tissue infections
IL-17RA deficiency	IL17RA	AR	Afflicted patients may also be at risk for *S. aureus* skin and soft tissue infections
IL-17RC deficiency	IL17RC	AR	
ACT1 deficiency	ACT1	AR	Afflicted patients may also be at risk for *S. aureus* skin and soft tissue infections
JNK1 deficiency	JNK1	AD	Syndrome with features of Ehler–Danlos-like connective tissue disorder
APECED	AIRE	Typically AR	Endocrinopathies. Neutralizing auto-antibodies to IL-17 and IL-22

## Data Availability

All data pertaining to the report are included within the manuscript.
